# The mineralization of osteonal cement line depends on where the osteon is formed

**DOI:** 10.1093/jbmrpl/ziaf114

**Published:** 2025-07-08

**Authors:** Astrid Cantamessa, Stéphane Blouin, Maximilian Rummler, Andrea Berzlanovich, Richard Weinkamer, Markus A Hartmann, Davide Ruffoni

**Affiliations:** Mechanics of Biological and Bioinspired Materials Laboratory, Department of Aerospace and Mechanical Engineering, University of Liège, 4000 Liège, Belgium; Trauma Centre Meidling, 1st Medical Department Hanusch Hospital, Ludwig Boltzmann Institute of Osteology at Hanusch Hospital of OEGK and AUVA, 1120 Vienna, Austria; Department of Biomaterials, Max Planck Institute of Colloids and Interfaces, 14476 Potsdam, Germany; Center of Forensics Medicine, Medical University of Vienna, 1090 Vienna, Austria; Department of Biomaterials, Max Planck Institute of Colloids and Interfaces, 14476 Potsdam, Germany; Trauma Centre Meidling, 1st Medical Department Hanusch Hospital, Ludwig Boltzmann Institute of Osteology at Hanusch Hospital of OEGK and AUVA, 1120 Vienna, Austria; Mechanics of Biological and Bioinspired Materials Laboratory, Department of Aerospace and Mechanical Engineering, University of Liège, 4000 Liège, Belgium

**Keywords:** cement line, mineralization, osteon, human bone, quantitative backscattered electron imaging, mineralization process, mineral content, mineral recycling, tissue age

## Abstract

The cement line (CL) is a thin layer, 1-3 μm in width, separating secondary osteons from interstitial bone and other osteons. Despite the possible role for bone quality, the CL is still one of the least understood features of bone. This study aims to investigate how the mineral content of the CL varies not only with osteon age but also with the surrounding environment. Using quantitative backscattered electron imaging to measure the mineral content, we analyzed 35 osteons from femoral bone of 2 male individuals (40 and 81 yr old). We implemented a new approach to investigate the mineral content based on a spatially resolved analysis in layers along the CL and incorporating regions both inside the osteon (formed soon after CL deposition) and outside (already present at the time of CL deposition). We found that the CLs had always higher mineral content than the corresponding osteon (*p* < .001) and that not only the osteon, but also the CL increases its mineral content with time. Including areas outside the osteon in the analysis improved considerable our understanding of CL mineralization. After a rapid primary phase, where the CL incorporates more mineral than the osteon, secondary mineralization is about 60% lower in the CL than in the osteon. One key finding is that the mineralization of the CL is not universal but depends on the region in which the osteon is formed. This is supported by a strong correlation between the mineral content of the CL and outside the osteon (*R* = 0.75, *p* < .001), but not inside. One possible explanation is that mineral released during bone resorption may contribute to the mineralization of the CL, as higher mineral content in resorbed bone was associated with greater mineralization in the CL.

## Graphical Abstract

**Graphical Abstract:**
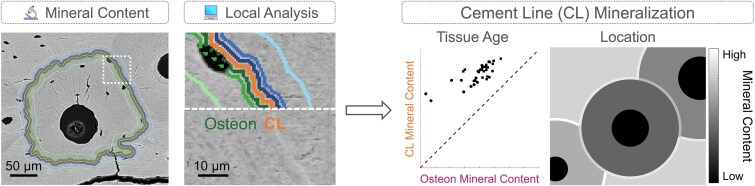

## Introduction

Cement lines (CLs) are thin interphases, typically 1-3 μm in width, which are ubiquitous in bone. They are found at: (1) the border of secondary osteons in cortical bone,^1^ (2) around individual bone packets in trabecular bone,^2^ and (3) between bone and mineralized cartilage at entheses^3^^,^^4^ and osteochondral junctions.^4^ Despite their small thickness, CLs are assumed to contribute to bone fracture resistance by deflecting or arresting microcracks. In cortical bone, studies have shown that microcracks originating from the interstitial tissue can deviate when meeting the CL.^5–8^ Such a mechanism allows to dissipate energy, to reduce the force driving crack propagation,^9^ and also to preserve the overall integrity of the osteon. Indeed, microcracks are more likely observed in interstitial bone than inside osteons.^10^

The ability of the CL to interact with damage depends on its basic mechanical properties, such as stiffness, strength, and toughness.^11^ However, these properties are still not well characterized: there are only very limited data on CL stiffness and strength^12^ and, to our knowledge, no data on fracture toughness. Cement line mineral content is the one factor of fundamental importance for the mechanical behavior. Yet, the composition of CLs remains controversial: both lower^13^ and higher^14^ mineral content of CLs compared to surrounding bone are reported. More recent data indicate that CLs tend to have higher mineral content than the corresponding osteons,^10^^,^^15^^,^^16^ with the difference decreasing with tissue age.^16^ Complementary Raman analysis has suggested that the higher degree of mineralization of the CL is combined with lower collagen content.^16^ At the same time, the dimensions of the osteon do not seem to influence the degree of mineralization of the CL.^16^

The mineral content of the osteon is not static but evolves over time according to a specific mineralization process. Current understanding is that a fast primary mineralization, bringing the mineral content up to ~70% of the final value within a few days, is followed by a much slower secondary mineralization. The latter leads to a gradual maturation of the mineral phase, which takes up to several years.^17–20^ Conversely, knowledge about how much mineral is incorporated during CL formation, and to which extent the mineral content of the CL evolves with time is insufficient.

Cement lines are found around secondary osteons, which are a result of cortical bone remodeling,^21^ with CLs providing a proper “cleaned” surface for osteoid deposition.^1^^,^^22^^,^^23^ Cement lines have higher content of osteopontin (a CL marker) than adjacent bone.^23^ The examination of cutting cones in osteons with serial sectioning has provided clues that in bone remodeling both osteoclasts and osteoprogenitor cells are able to form CLs during or immediately after bone resorption.^1^ In bone modeling, however, CLs are laid down on surfaces not previously eroded, and only osteoprogenitors cells should be contributing to their formation.

A further element that can impact tissue mineralization is the transport of mineral ions to the mineralization site. There is evidence that locations of bone modeling and remodeling exhibit differences in the mineralization process, resulting in specific mineral characteristics, such as crystal composition and dimensions.^24^ This is likely due to different avenues to transport ionic precursors. Usually bone modeling requires transport over large distances and, thus, ionic precursors should be stabilized by compositional modifications, whereas in bone remodeling this is not necessary as resorbed mineral may be locally recycled.^24^ As the formation of CL and osteonal bone are somewhat dephased, both in space and time, differences in available mineral ions may be present, impacting the mineralization process.

The aim of this work is to characterize how the mineral content of CLs varies with the surrounding environment and changes over time. By examining osteons of different mineral content, potentially reflecting differences in tissue age, new insights into CL mineral content and mineralization can be obtained.

## Materials and methods

### Sample preparation

**Figure 1:**
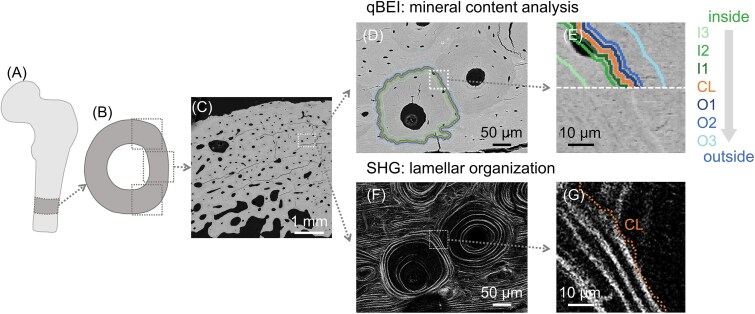
Analysis of human osteonal bone. (A) The schematic shows a human femur and (B) transverse cross-section where (C) qBEI scans were performed to select the osteons to be analyzed. (D) High-resolution qBEI map of an uninterrupted osteon neighboring other osteons (top and bottom) and interstitial bone (left and right). (E) Magnified view of a CL and the different layers around the CL, located both inside (I1, I2, I3) and outside (O1, O2, O3) the selected osteon. (F) SHG image of the same region highlighting the uninterrupted lamellar organization of the selected osteon. (G) Close-up of the SHG image with the orange dashed line representing the location of the CL, detected by superimposing SHG and qBEI maps.

Human femurs from 2 male donors, including a 40-yr-old individual (height 175 cm, weight 77 kg, cause of death myocardial infarction) and a 81-yr-old individual (height 175 cm, weight 79 kg, cause of death myocardial infarction) without known metabolic bone disease, were obtained from the Department of Forensic Medicine of the Medical University of Vienna, in accordance with the ethical commission of the institution (EK no. 1757/2013). After extraction, the bones were kept frozen at −20 °C until further preparation. Cross sections (perpendicular to the longitudinal axis of the femurs) of about 1.5 cm in thickness were cut from the femoral shaft far from the metaphysis (Figure 1A and B) using a water-cooled diamond saw (Buehler Isomet 1000). The sections were then prepared following a well-established procedure,^25^^,^^26^ which involved dehydration in graded ethanol series, followed by immersion in ethanol/acetone baths, and embedding in poly-methyl methacrylate. The transverse surfaces were ground with sandpapers having decreasing grit size (P1200, P2400) and polished using a diamond suspension down to 1 μm particle size on a silk cloth under glycol irrigation (Logitech PM5), to minimize the formation of microcracks.^27^

### Quantitative backscattered electron imaging (qBEI)

The local mineral content of cortical bone was quantified with qBEI, a well-established technique based on SEM, providing quantitative two-dimensional spatial maps of calcium content by measuring the intensity of the backscattered signal.^26^^,^^28^ To obtain a conducting surface, a thin carbon layer was deposited on the polished samples by vacuum evaporation (Agar SEM carbon coater, Agar). The SEM, equipped with a zirconium-coated field emission cathode (Zeiss SEM SUPRA 40), was operated at 20 kV with a working distance of 10 mm, and a probe current of 280-320 pA. First, overview scans (Figure 1C) with a larger field of view and a lower resolution (1.76 μm/pixel) were performed to select the osteons of interest for subsequent higher resolution analysis. The selected osteons had to feature a central Haversian canal and a continuous border, that is, they should not be interrupted by more recent osteons. In the analysis, we included osteons with different sizes and mineral contents, which is assumed as a proxy of tissue age. The chosen osteons (*n* = 35, 15 coming from the younger donor and 20 from the older one) and the surrounding bone were measured at higher magnification (pixel resolution 0.57 μm with a field of view of 1024 × 768 pixels). This resolution is high enough to resolve the CLs, which have a thickness in transverse sections of about 1-3 μm and low enough to allow for a quantitative measurement of the calcium content without additional contrast due to, for example, orientation of bone lamellae. A calibration of the SEM backscattered signal with carbon and aluminum standards was performed as previously reported,^26^^,^^29^ to establish a quantitative relation between the measured gray level and the local mineral content, expressed as calcium weight percent (Ca wt%).

### Second harmonic generation imaging

Second harmonic generation (SHG) imaging was performed at the same locations analyzed with qBEI to ensure that the selected osteons are the most recently deposited in their neighborhood. This can be challenging when 2 osteons with similar mineral content slightly overlap (Figure 1D). By inspecting the lamellar organization accessible with SHG (Figures 1F and G and S1), we look for osteons with uninterrupted lamellae, which is a clear indication of being the most recent ones. Samples were imaged using confocal laser scanning microscopy (Leica TCS SP8 DLS) with a 40× oil immersion objective (HC PL APO 40×/1.30 OIL). A pulsed infrared laser (Spectra-Physics) at a wavelength of 910 nm was used, and the backward direction signal was detected at 450-460 nm wavelengths. Images were acquired with a nominal pixel size of 379 nm and a field of view of 1024 × 1024 pixels at a scan rate of 400 Hz. Several neighboring regions were measured with a slight overlapping, ensuring proper alignment when stitching them together using the software of the Leica microscope. For an appropriate detection of lamellar arrangement, the SHG resolution was chosen to be smaller than typical lamellar thickness ($\sim$4-6 μm).

### Mineral content of the CL and adjacent regions: layers analysis

Before evaluating the mineral content, a 2-pixel erosion was applied to minimize boundary effects^17^ using the software CTAn (v1.19.4.0, Skyscan). Background pixels, including osteocytes lacunae, Haversian canals and microcracks, were removed by discarding all pixels with a calcium content below 5.2 wt%. This ensured that only mineralized bone regions and no voids were included in the analysis. The mineral content of the CL and of neighboring bone was evaluated according to the following layer analysis, implemented in CTAn. First, a mask of each osteon (not including the CL) was generated by manually contouring the osteon border as delimited by the CL (Figure S2A and B). This choice is motivated by the fact that the boundary between the CL and the osteon was usually easier to detect than between the CL and the interstitial bone. The manual contouring process was performed by a single operator to maintain consistency, and the results were visually inspected by a second operator. The extracted mask was dilated with a round kernel of 2 pixels to include the CL, and the original mask of the osteon was subtracted from the dilated mask to obtain a 2-pixel (or 1.14 μm) thick circular layer representing the CL. Next, the bone around the CL was subdivided into several concentric circular layers having the same nominal thickness of the CL and located both inside and outside the osteon, in regions adjacent to the CL as well as further away (Figure 1D and E). The layers were obtained by multiple cycles of erosion and dilation applied to the mask of the osteon (Figure S2C and D). Specifically, we considered layers close to the CL and placed at 1 and 4 pixels (or 0.57 μm and 2.28 μm) away from the CL border: 2 layers inside the osteon (I1, I2) and 2 layers outside (O1, O2). Two additional layers were defined 20 pixels (or 11.4 μm) away from the CL (I3 and O3, respectively). Inner layers include only osteonal bone, whereas outer layers may intersect with other (older) osteons and interstitial bone. Furthermore, all concentric circular layers were radially sliced into 16 sectors (using MATLAB 2022a), which served to investigate the spatial variation of mineral content around the osteons (Figure S2E). A custom MATLAB routine was implemented to compute the mean calcium content (Ca_Mean_), its SD, as well as the frequency distribution of the calcium content, within each layer and in the whole osteon. Considering the limited number of pixels in the layers, to improve readability of the plots, all frequency distributions were slightly smoothed with a running average filter (window size 10).

### Statistical analysis

We compared differences in mineralization among the layers by performing a Friedman test, chosen due to the non-normal distribution of the paired data. When significant differences were detected, pairwise comparisons between layers were conducted using post-hoc tests with Bonferroni correction to control for multiple comparisons and minimize false positive. We examined the strength of the relationships between the mean calcium content of osteon, CL and different layers using Spearman’s rank correlation, selected for its suitability with non-normal distribution of the data. Significance levels were set at *p* < .05, and all *p*-values were two-sided. All statistical analyses were performed in MATLAB 2022a.

## Results

From a qualitative observation of the qBEI maps, the CL appears as a thin line around the osteon (Figure 1E). In the SHG images, there were no evident features allowing a clear discrimination between CL and adjacent lamellar bone (Figure 1G). However, superimposing qBEI and SHG maps indicates that the CL tends to fall in regions with no (or very weak) SHG signal.

We explore possible links between the mineralization of the CL and immediately adjacent bone, considering regions both outside and inside the osteon, that is, formed before and after CL deposition, respectively. We first report detailed findings on 2 osteons having low and high mean mineral content that are representative for younger and older tissue (assuming mineral content as an indirect measure of tissue age) and extracted from the adult (40 yr old) and aged (81 yr old) individual, respectively. We then present results of all 35 osteons, with mean calcium content ranging from 21.37 ± 2.26 wt% to 26.43 ± 2.2 wt%, and therefore covering a large spectrum of tissue age. Less mineralized younger osteons were more likely found in the adult individual, whereas older more mineralized osteons were found in the aged individual.

**Figure 2:**
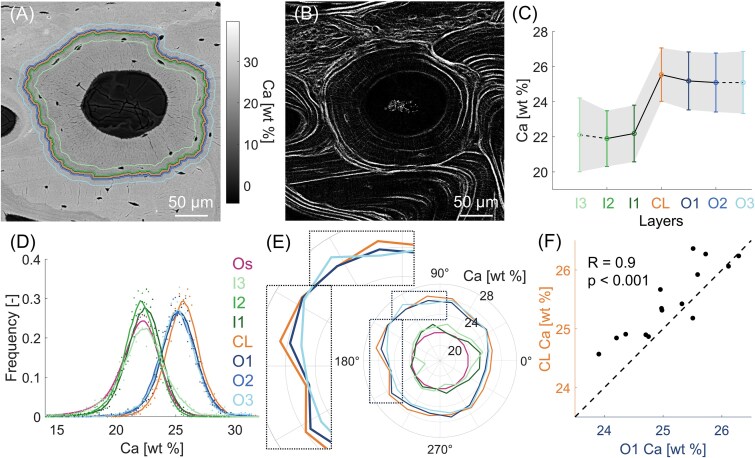
Mineral content analysis of a lowly mineralized osteon from the adult (40 yr) individual. (A) qBEI map with CL and layers highlighted by the colored lines. (B) SHG image showing the lamellar organization. (C) Mean calcium content of the inner layers (I3, I2, I1), CL and outer layers (O1, O2, O3). Data shown as mean ± SD (the shaded area delimits a one-SD interval around the mean). Data points are connected by lines to visualize the trend: full lines connect points equally spaced, whereas dashed lines indicate that I3 and O3 are further away. (D) Frequency distributions of the calcium content of the entire osteon (Os), CL and layers. Original data represented as scattered points and smoothed data (with moving average) as full lines. (E) Polar plot of the calcium content in selected regions (CL, O1, O3, I1, I3, and Os), averaged over 16 sectors around the osteon. The mean Ca content in each sector is plotted as distance from the center. (F) Correlation between the calcium content of CL and O1 considering all 16 sectors. The dotted line with a unit slope and passing through the origin is shown to improve the readability of the plot.

As a first example, we investigated an osteon with relatively low mean mineral content (Ca_Mean_ = 21.72 ± 2.09 wt%) coming from the adult individual and surrounded by older osteons and interstitial bone (Figure 2A). Second harmonic generation imaging reveals bone lamellae at the osteon periphery, confirming that the selected osteon is not interrupted by other osteons (Figure 2B). The local and layer-based analysis of qBEI map indicates a clear increase in mineral content of about 15% when going from the inner layers of the osteon to the CL, which has Ca_Mean_ of 25.54 ± 1.52 wt% (Figure 2C). The difference in mineral content between the CL and the outer layers is much smaller (around 2%) but still statistically significant (*p* < .001). The same trend is confirmed when inspecting the frequency distributions of the calcium content of the layers, which essentially split into 2 groups (Figure 2D): inner layers have similar distributions, overlapping with the distribution of the whole osteon, whereas the distributions of the outer layers are shifted toward higher mineral content. The frequency distribution of the CL displays an additional (albeit small) right-shift, making it distinguishable from all other curves. Owing to the circular shape of the osteon, the spatial correlation among the layers is further explored with a polar plot (Figure 2E), based on the subdivision into 16 sectors (see Materials and methods). In the polar plot, the mean calcium content in each sector is plotted as distance from the center. If 2 layers have similar calcium content in the same sector, the corresponding polar plots overlap. A central observation is that in each sector around the osteon, the mean calcium content of the CL follows very closely the mineral content of immediately adjacent bone located just outside the osteon and therefore deposited potentially long before the CL. The polar plot also gives an idea of how the spatial correlation varies across the 16 sectors: for example, when the osteon is close to interstitial bone with higher mineral content (top region of the plot), the mineral content of both the CL and the outer layer O1 slightly increases. An additional quantification is obtained by computing the Spearman correlation coefficient between the calcium content of the CL and of the adjacent outer layer O1 in the 16 sectors around osteon (Figure 2F): with *R* = 0.9 (*p* < .001), such correlation can be considered as very strong.^30^ Conversely, no spatial relationship between the mean mineral content of CL and inner layers is evident: the correlation between the calcium content of the CL and of the inner layer I1 in the 16 sectors is not statistically significant (Figure S3A, *R* = −0.4, *p* = .108).

**Figure 3:**
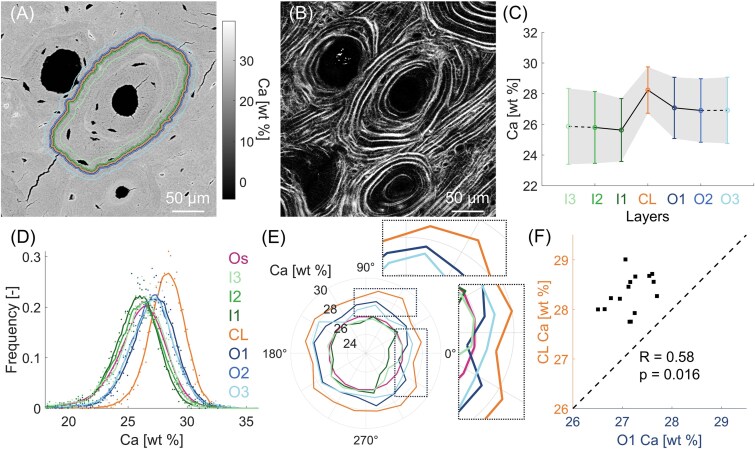
Mineral content analysis of a highly mineralized osteon from the aged (81 yr) individual. (A) qBEI map with CL and layers highlighted by the colored lines. (B) SHG image showing the lamellar organization. (C) Mean calcium content of the inner layers (I3, I2, I1), CL and outer layers (O1, O2, O3). Data shown as mean ± SD (the shaded area delimits a one-SD interval around the mean). Data points are connected by lines to visualize the trend: full lines connect points equally spaced, whereas dashed lines indicate that I3 and O3 are further away. (D) Frequency distributions of the calcium content of the entire osteon (Os), CL and layers. (E) Polar plot of the calcium content in selected regions (CL, O1, O3, I1, I3, and Os), averaged over 16 sectors around the osteon. The mean Ca content in each sector is plotted as distance from the center. (F) Correlation between the calcium content of CL and O1 considering all 16 discrete sectors. The dotted line with a slope of one and passing through the origin is shown to improve the readability of the plot.

The second example is an osteon with higher mineral content (Ca_Mean_ = 25.95 ± 2.45 wt%) representative for older tissue and extracted from the aged individual. Again, this osteon is surrounded by a heterogeneous environment featuring older osteons as well as interstitial bone (Figure 3A and B). The SHG analysis underlines a clear pattern of alternating and uninterrupted bone lamellae within the selected osteon (Figure 3B). In this “aged” scenario, the CL has still higher mineral content than the osteon (about 9%) as well as than the external surrounding bone layers (about 5%, Figure 3C). The frequency distributions of inner and outer layers are closer to each other with respect to the previous case of the younger osteon, whereas the distribution of the CL still displays a noticeable shift to higher mineral content (Figure 3D). The polar plot suggests a statistically significant spatial relationship between the mineral content of CL and adjacent outer layers (Figure 3E), which is characterized by a moderate correlation coefficient (*R* = 0.58, *p* < .05). Also in this example, the correlation between the CL and the inner layer I1 is not significant (Figure S3B, *R* = 0.41, *p* = .101).

**Figure 4:**
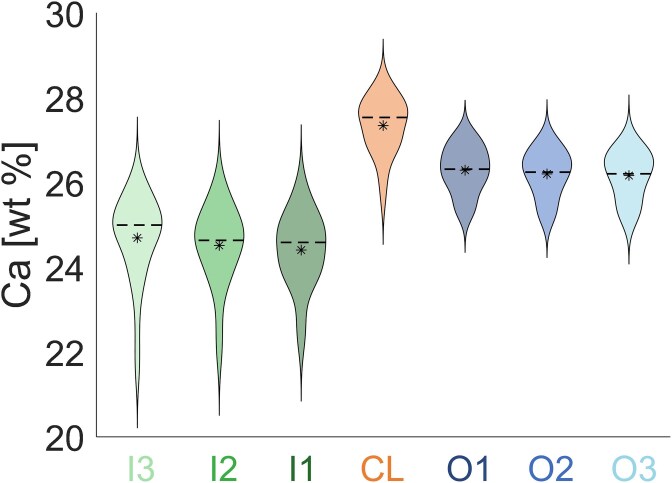
Violin plots (mirrored histograms) of the calcium content for each segmented region (CL, inner, and outer layers) considering all analyzed osteons (*n* = 35). The mineral content of the CL is significantly higher (*p* < .001) than all other layers but O1. The median and mean values are indicated by dashed lines and asterisks, respectively.

Extending the layer analysis to all osteons (Figure 4), confirms that the CLs always exhibit the highest mean mineral content (27.35 ± 1.58 wt% Ca), not only in comparison with the layers inside the osteons but also with the layers outside, apart from O1. The difference in mineral content between the CL and the outer layers is less pronounced than between the CL and the inner layers. The outer layers show a smaller spreading in the mean mineral content when compared to the inner layers.

**Figure 5:**
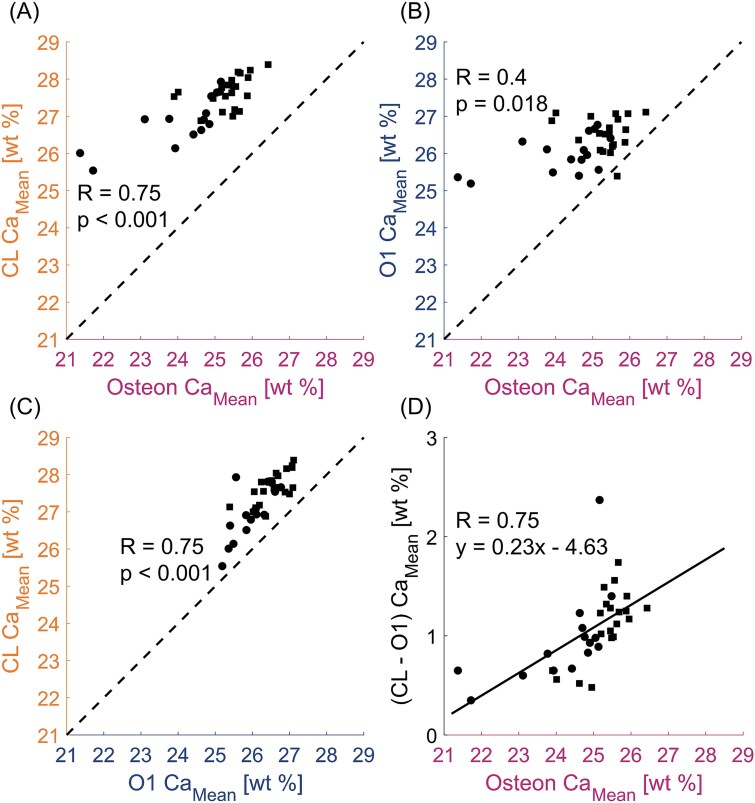
Relationship between CL, osteon and the outer bone. (A) Correlation between average calcium content (Ca_Mean_) of the CL and the corresponding osteon, (B) outer layer O1 and osteon, (C) CL and O1, and (D) difference between CL and O1 (CL − O1) and osteon. The dotted line (with a unit slope and passing through the origin) is inserted to improve the readability of the plot. The full line in (D) represents the result of a linear regression. Circles represent osteons from the adult individual (40 yr) and square from the aged individual (81 yr).

The mineralization of the CL is explored in Figure 5. First, we observed that the mineral content of the CL is always higher than the osteon mineral content and that a strong correlation is present between them (*R* = 0.75, *p* < .001, Figure 5A), suggesting that the CL mineral content progresses with tissue age. At the same time, the mineral content of the bone outside the osteon (represented by the outer layer O1) had only a moderate correlation (*R* = 0.4, *p* = .018) with the mineral content of the osteon (Figure 5B). In contrast, the relationship between the mineral content of CL and outer layer O1 (Figure 5C) displays a strong correlation (*R* = 0.75) with statistical significance (*p* < .001). As in Figure 5A, all experimental points are above the line with a slope of 1 (ie, 45°, dashed lines in Figure 5A-C). This confirms that the CL is the highest mineralized structure in all 35 investigated osteons compared to either the inside or the outside of the osteon. Virtually identical results are found when considering instead of the outer layer O1, the outer layer O2, which is further away from the CL (Figure S4A and B). The strong correlation between the mineral content of the CL and the outer layer O1 motivates to investigate how the difference between these 2 quantities (CL − O1) behaves when plotted vs the osteon mineral content (Figure 5D). The obtained correlation is strong (*R* = 0.75) and statistically significant (*p* < .001). The positive slope of the correlation (full line in Figure 5D) shows that the mineral content of the CL deviates more from the mineral content of the surrounding, the older (ie, the higher mineralized) the osteon is. An opposite trend is observed when plotting (CL − I1) vs the osteon mineral content (Figure S5).

**Figure 6:**
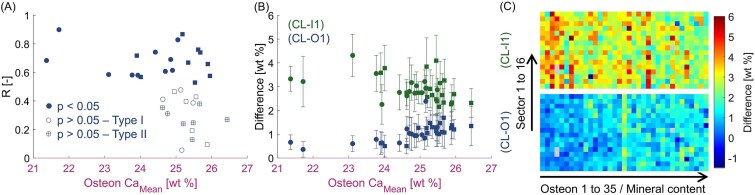
Spatial analysis of the mineral content. (A) Spearman correlation coefficients between the mineral content of the CL and the outer layer O1. All osteons that show a statistically significant correlation are of type I, whereas among the osteons where no correlation is detected, there are several osteons of type II (ie, osteon-in-osteon). (B) Differences in mineral content between the CL and the neighboring layers computed as average difference (CL − O1) and (CL − I1) in the circular sectors and plotted vs the mean degree of mineralization of the osteon. Data points represent the mean value and error bars the SD for each osteon. In (A) and (B), circles represent osteons from the adult individual (40 yr) and square from the aged individual (81 yr). (C) Local differences (CL − O1) and (CL − I1) calculated in each sector (vertical axis) and reported for each osteon sorted according to increasing mineral content (horizontal axis). A few gray pixels indicate sectors, where the mineral content could not be calculated, for example, due to pores or cracks.

Motivated by the strong correlation between the mineral content of the CL and of the outer layer O1 (Figure 5C), we performed a spatially resolved analysis of the relationships between the mineralization of the CL and the outer/inner layers, by considering the calcium content within each circular sector around the osteon, for a total of 3920 different regions inspected (Figure 6). Pooling all sector-wise data together (Figure S6) confirmed the trend observed in Figure 5, with CL and O1 showing the highest correlation and data points spread close to a line of unit slope. We applied the sector analysis (presented in Figures 2F and 3F) to all osteons (Figure 6A): for 21 out of 35 osteons, we detected a statistically significant correlation (*p* < .05) between the mineral content of the CL and outer layer O1, ranging from moderate (0.4 < *R* < 0.7) to strong (0.7 < *R* < 0.9). The osteons which did not show a significant correlation are likely to have higher mineral content (Ca_Mean_ > 24.6 wt%). The differences in mineral content between the CL and the closest outer layer (CL − O1) as well as between the CL and the closest inner layer (CL − I1) were then calculated in each discrete sector and averaged for each osteon (Figure 6B). The results further underline that the degree of mineralization of the CL is extremely close to the mineral content of the outer layer O1, with the difference CL − O1 being always less than 2 wt% and showing a small increase for more mineralized osteons. In comparison, the differences between CL and I1 are higher (up to 3.5-4 wt%) and show a slight decreasing trend with osteon mineral content. A similar behavior is observed when considering all individual sectors (Figure 6C). Here, we report results as 2D maps with differences (CL − O1) and (CL − I1) calculated in each sector but not averaged in the osteons. In Figure 6C, the 16 individual sectors are on the vertical axis and the 35 osteons, sorted from lower to higher mineral content, are on the horizontal axis. Results indicate that even in discrete regions around the osteon, the mineral content of the CL is very similar to the degree of mineralization of the outer layer O1; this similarity being particularly strong for osteons with lower mineral content, that is, assumed to be younger.

## Discussion

In this study, we investigated the mineral content of CL and neighboring tissue in cortical bone, and we deduced the following aspects of CL mineralization.

The observations that the mineral content of the CL is higher compared to the corresponding osteon and that there is a strong correlation between the mineral content of the CL and of the osteon (Figure 5A), suggest that the mineral content of the CL also evolves in time. To describe the mineralization process, a separation into a fast primary and a much slower secondary phase is useful. Osteon and CL have a similar tissue age. Nevertheless, the higher mineral content of the CL suggests that in primary mineralization, the CL can incorporate more mineral than the osteon. A previous study on postmenopausal women reported similar trends.^16^ However, our work demonstrates that more can be learned when the focus is not restricted to the osteon itself and the outer environment around the CL is explored. Our main finding is that the mineral content of the CL (after primary mineralization) is closely related to the mineral content of the surrounding in which the CL and the new osteon are formed. This statement is supported by the strong correlation between the average mineral content of the CL and of the external bone (Figure 5C). Noteworthy, a correlation is even present when considering individual regions around the osteons (Figure 6), hinting for a local spatial interplay between the older resorbed bone CL and the newly deposited CL. How can such an interplay be explained? A possible explanation should consider the formation of the CL, which takes place on the eroded surface soon after bone resorption and therefore in a local milieu full of mineral ions released by osteoclasts,^31^^,^^32^ with the additional presence of osteopontin, that could further enhance the accumulation of minerals in the newly forming CL.^33^^,^^34^ It is conceivable that the higher the mineral content of the region undergoing resorption, the more mineral ions are released and used to build the CL. This scenario is compatible with a faster primary mineralization of the CL, explaining also the strong correlation between the mineral content of the CL and of layer O1 (Figure 5C). As this correlation is higher than the one between osteon and O1 (Figure 5B), secondary mineralization alone cannot be the only cause for the observed trend. The formation of the bone inside the osteon is somewhat “dephased,” both in space (up to 1 mm^1^) and time (up to 50 d, assuming a deposition rate of 20 μm/d^35^), with respect to the deposition of the CL. Thus, the concentration of mineral ions during osteonal bone deposition may be different than during the formation of the CL. This could be one reason of the weak correlation between CLs and bone layers inside the osteon (Figure S7).

Considering secondary mineralization, Figure 5D clearly shows that the difference between the mineral content of the CL and of its outside surrounding increases with tissue age. Since the surrounding of the selected osteons is older bone tissue, which is already in advanced secondary mineralization, it can be assumed that its mineral content hardly changes any more. Consequently, this increasing trend should correspond to the rather slow secondary mineralization of the CL. Based on available data on turnover rate in osteonal bone,^36–38^ we estimate an age difference of about 15 yr between the youngest and the oldest osteon. This means that the mineral content of the CL increases due to secondary mineralization of about 2 wt% over 15 yr. In contrast, within the same time span, the increase in osteon mineral content is about 5 wt% (Figure 5A). A secondary mineralization, which is slower in the CL compared to the osteon, is also consistent with the finding that the higher the mean mineral content of the osteon, the less is the difference in mineralization between the osteon and the CL (Figure S8).

In some cases, the correlation between the mineral content of the CL and the outer surrounding is weak, and we could identify a reason for that: many of the osteons with a weak correlation are classified as type II osteons^39–41^ also known as “osteon-in-osteon”^42^ (Figure 6A). This term refers to a specific type of relatively large osteon containing a smaller osteon in its center, both being surrounded by concentric CLs.^39^^,^^43^ In type II osteons, the degree of mineralization of the external environment around the osteon tends to be more homogenous in comparison to osteons surrounded by both interstitial bone and older osteons (Figure S9).

In summary, we provide information on the mineralization process of the CL: after primary mineralization, the mineral content of the CL is substantially higher than the mineral content in osteonal bone. Most importantly, the mineral content of the CL after primary mineralization is clearly not universal but depends on the location where the remodeling event occurs, with the CL being more mineralized when the osteon is surrounded by bone of higher mineral content. The CL shows also a secondary mineralization, which proceeds at much slower rates than in osteonal bone. One reason for the slower pace may be the high mineral content already attained at the end of primary mineralization. The interplay between primary and secondary mineralization of the CL can be explored with the help of a previously developed mathematical model for the mineralization process of bone^18^ (Supplementary Information and Figure S10): if the CL has an augmented primary mineralization, the secondary mineralization needs to be reduced to avoid attaining unrealistically high values of the mineral content (Figure S10). The found higher mineral content of all CLs compared to the mineral content of the neighboring osteonal bone agrees with previous works^10^^,^^14^^,^^16^ and one possible explanation is a reduced nanoscale porosity at the CL.^25^

The following limitations should be mentioned. A major point concerns the limited sample size of only 2 male individuals and 35 osteons, mainly because of the time-consuming approach of our analysis. Nonetheless, our observations on the interplay between CL and osteon mineral content are in full agreement with a prior work,^16^ corroborating that these findings are not unique to the 2 individuals studied. Future works should incorporate larger and more diverse samples, coming from individuals of different gender, age, as well as considering different anatomical locations. We have selected males for this first investigation to rule out the possible impact on bone mineral content following menopause in women.^47^ Another point is about the limited thickness of the CL. We acquired qBEI maps with a resolution of 0.57 μm/pixel and the typical width of the CL in 2D sections is about 1-3 μm. Consequently, there may be a fraction of pixels only partially filled by the CL, leading to partial volume artifacts impacting the detected mineral content. We tried to minimize this issue by considering a 2-pixel layer centered on the CL. While this approach likely underestimates the true thickness of the CL around each osteon in most cases, it provides a conservative estimation of its mineral content. In our work as well as in the literature,^16^^,^^24^^,^^44^^,^^45^ the osteon mineral content is used as an indicator of tissue age. Although we cannot exclude that mineralization progresses at different speeds in different osteons, the mineral content remains a valuable quantity for studying mineralization in the absence of direct tissue age markers.^46^ Although improbable, it cannot be completely ruled out that freezing and treatment of samples affects the measured mineral content. Nevertheless, all samples were prepared in the identical way, allowing for comparisons between osteons and ensuring validity of found correlations. While this work focuses on mineral content and mineralization, future works should characterize the organic phase for example with Raman microspectroscopy. Such an analysis would give the possibility to detect possible changes in the organic matrix, for example, the glycosaminoglycan content that might contribute to the observed differences in mineralization.

In conclusion, considering the bone outside the osteon, we demonstrate a close spatial interplay between the mineralization of the CL and the mineral content of the surrounding older bone, suggesting that CL mineralization is different in different osteons. Bone diseases or treatments affecting bone mineralization may have a direct impact on the mineral content, and therefore on the mechanical behavior of the CLs.

## Supplementary Material

JBMRp_Cantamessa_SI_ziaf114

## Data Availability

The datasets generated and analyzed during the current study are available from the corresponding authors on reasonable requests.
